# A pheromone outweighs temperature in influencing migration of sea lamprey

**DOI:** 10.1098/rsos.150009

**Published:** 2015-05-06

**Authors:** Cory O. Brant, Ke Li, Nicholas S. Johnson, Weiming Li

**Affiliations:** Department of Fisheries and Wildlife, Michigan State University, Room 13 Natural Resources Building, 480 Wilson Road, East Lansing, MI 48824, USA

**Keywords:** pheromone, temperature, migration, sea lamprey, cue

## Abstract

Organisms continuously acquire and process information from surrounding cues. While some cues complement one another in delivering more reliable information, others may provide conflicting information. How organisms extract and use reliable information from a multitude of cues is largely unknown. We examined movement decisions of sea lampreys (*Petromyzon marinus* L.) exposed to a conspecific and an environmental cue during pre-spawning migration. Specifically, we predicted that the mature male-released sex pheromone 3-keto petromyzonol sulfate (3kPZS) will outweigh the locomotor inhibiting effects of cold stream temperature (less than 15°C). Using large-scale stream bioassays, we found that 3kPZS elicits an increase (more than 40%) in upstream movement of pre-spawning lampreys when the water temperatures were below 15°C. Both warming temperatures and conspecific cues increase upstream movement when the water temperature rose above 15°C. These patterns define an interaction between abiotic and conspecific cues in modulating animal decision-making, providing an example of the hierarchy of contradictory information.

## 2. Introduction

Environmental cues (e.g. abiotic information) and signals (e.g. an entity evolved by a sender that elicits an evolved behavioural response in a receiver) provide a constant input of information used by organisms in decision-making processes [1,2]. Exactly when certain information becomes more reliable in the decision-making process is probably shaped by natural selection (e.g. decisions that ultimately impact the recipient's fitness) [2]. How organisms distinguish reliable information from a multitude of environmental, conspecific and heterospecific cues remains largely unknown.

Among abiotic cues, water temperature is critical in influencing the timing of fish migration [3]. Pheromones [4,5] are signals that may inform fish of mate readiness and/or habitat suitability [5]. The sea lamprey relies on environmental and conspecific cues during reproduction. Specifically, when water temperatures drop below 15°C, sea lamprey become increasingly less active. Warmer water temperatures correlate with increases in locomotor activity of pre-spawn migrating sea lamprey, probably as an adaptation to reach spawning grounds before full maturation of gametes occurs [6]. In addition, the odour of stream-resident larvae guides pre-spawning sea lamprey in selecting suitable spawning habitat [7,8]. Later, sexually mature female sea lamprey rely on a sex pheromone released by mature males (3-keto petromyzonol sulfate, 3kPZS) [9] to locate nesting mates [10].

We hypothesized that the conspecific male sex pheromone (3kPZS) contains more reliable information compared with water temperature regarding spawning conditions upstream. Here, we report evidence that both male and female pre-spawn adults move upstream in the presence of 3kPZS within the otherwise locomotor inhibitory temperature range (below 15°C), which is consistent with the hypothesis. While test subjects move upstream, they do not bias towards a channel-side containing the source of 3kPZS during our studies, a previously described behaviour in mature female conspecifics [9,10].

## 3. Material and methods

### 3.1 Animals and tagging

Procedures involving sea lamprey were approved by the Michigan State University Institutional Animal Care and Use Committee (AUF no. 05/09-088-00). Immature adult sea lamprey were captured by the United States Fish and Wildlife Service and Fisheries and Oceans Canada from tributaries to Lake Michigan and Lake Huron, in May–June 2009, 2010 and 2012, and transported to the United States Geological Survey Hammond Bay Biological Station (HBBS) for further procedures. Sex and maturity determination, and animal housing followed procedures described previously [10]. Pre-spawn sea lamprey refers to river-migrating adults that are non-feeding, yet not fully sexually matured. Pre-spawn sea lamprey were implanted with a 23 mm long half duplex passive integrated transponder (PIT) tag (Oregon RFID, Portland, OR, USA) through a 3 mm lateral incision in the mid-abdominal region.

### 3.2 Pheromones

Use of 3kPZS (synthesized by Bridge Organics, Vicksburg, MI, USA; purity greater than 97%) in the stream was permitted by the United States Environmental Protection Agency (experimental user permit no. 75437-EUP-2). A 10 mg ml^−1^ stock solution of synthesized 3kPZS (in 50% methanol) was prepared. 3kPZS stock solution was stored at −80°C until use in the field. Larval extracts, extracts of water conditioned with larval sea lamprey [11,12], were used as a positive control to validate the experimental system [7,8]. To collect extracts of water conditioned with larval sea lamprey, over 20 000 larval sea lamprey were held in flowing 500 l-capacity tanks at HBBS from April to August 2008. Larvae were given a sand substrate for refuge and fed yeast weekly. Tank flows were shut off between approximately 20.00–08.00 hours (h), allowing larval odour to concentrate. Larval-conditioned water was passed through vertical columns containing 500 g of methanol-activated Amberlite XAD7HP resin (Sigma-Aldrich, St Louis, MO, USA) using peristaltic pumps (Masterflex 7553-70, Cole-Parmer, Vernon Hills, IL, USA). Loading speed was approximately 300 ml min^−1^. Three columns were loaded for up to 24 h at a time. Each column was then eluted with 4 l of methanol. Eluents were concentrated using a model R-210 roto-evaporator (Buchi Rotavapor, Flawil, Switzerland) and stored at −80°C. All larval extracts were fully thawed, pooled and thoroughly mixed before further analyses were conducted. Petromyzonamine disulfate (PADS), a component of larval extract [13], was used as a benchmark when calculating the volume of larval extract needed to activate the stream, with PADS at a concentration of 5×10^−14^ M. The concentration of PADS in the larval extract was determined using high-performance liquid chromatography–tandem mass spectrometry [12].

### 3.3 Field behavioural tests

**Figure 1.**
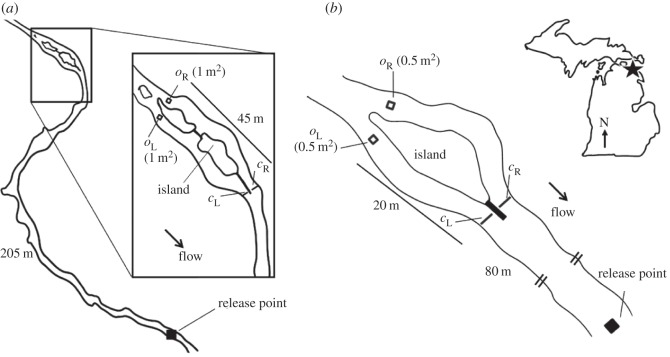
(*a*) Schematic of the Upper Ocqueoc River (Millersburg, MI, USA) field site for female testing only. (*b*) Schematic of Upper Trout River (Rogers City, MI, USA) field site for male testing only. Transecting right (*c*_*R*_) and left (*c*_*L*_) PIT antennas recorded the number of test subjects that moved into the treatment subchannel (alternated each trial). Square-frame *o*_R_ and *o*_L_ PIT antennas recorded animals entering near the treatment source.

Subjects were tested at night, when migrating sea lamprey move upstream [14], in experimental sites shown in figure 1. Barriers near each river-mouth prevent wild migrating sea lamprey from entering the upper reaches. Females were tested in a separate field site (figure 1*a*) than males (figure 1*b*) to prevent unwanted reproduction of this invasive species above each barrier. The most upstream section of each site was bifurcated by a natural island, which separated two subchannels of similar hydrological and physical qualities. Test odourants were diluted with 30 l of river water in large mixing bins. Bins were kept consistent for each test odourant to reduce the potential for contamination during dilution. Each solution was pumped into respective subchannels through separate latex tubes at a rate of 167 ml min^−1^ (±5 ml min^−1^) over the span of three hours using peristaltic pumps (Cole-Parmer). A test odourant was administered to one subchannel (activated channel, herein), whereas an equal volume of methanol was administered into the adjacent subchannel (control channel, herein), and the activated and control channels were alternated each trial.

Trials were conducted between 13 May and 11 June 2009, between 3 May and 8 June 2010 (females), and between 19 and 31 May 2012 (males), at night. Stream discharge was estimated every 3 days, or after every precipitation event, at a fixed location in the stream using a Marsh-McBirney portable flow meter (Flo-Mate 2000, Fredrick, MD, USA) to determine the amount of odourant stock solution to apply to the stream and maintain consistent concentrations across trials. Stream flow was relatively uniform within each site, and flows were lower on average in the male-only site (950–1200 m s^−1^ in figure 1*a*, and 200–320 m s^−1^ in figure 1*b*).

Up to two trials were conducted each night, depending upon animal availability. The early trial was conducted from sundown, approximately 20.30–23.20 h, and a late trial was then run from approximately 00.10 to 03.10 h (trial times were dependent upon when sundown occurred). Twenty or 30 PIT-tagged sea lamprey (depending on animal availability) were removed from holding tanks at HBBS and transported to their respective stream acclimation cage at the release point (figure 1) between 03.00 and 05.00 h the night prior to experimentation. Acclimation/release cages were mesh aluminium (approx. 0.25 m^3^) consisting of a sliding door that was removed manually upon release. Animals were then allowed an acclimation period in the stream for 15+ h. Mortality during acclimation was less than 0.5%.

Each trial was 3 h long. Stream temperature was recorded at the start of each trial. In the first hour of each trial, the test odourant was administered to the stream. At the start of the second hour, test animals were released. During the remaining 2 h, animal behaviours were monitored while odourants were administered. The second trial began 30 min after the first. Test odourants were kept consistent for each night of trials. As an example, if 3kPZS was tested during the early trial, then 3kPZS was also tested in the opposite subchannel during the late trial to prevent the possibility of any unwanted contamination from other test odourants. No animals were recovered from the stream after a trial. Each test subject's unique PIT tag identification number prevented any pseudo-replication from test subjects released during previous nights. Movement data were consolidated and stored using a multiplexor (Oregon RFID, Portland, OR, USA). Data were uploaded each trial night using a hand-held Meazura model MEZ1000 personal digital assistant (Aceeca International Limited, Christchurch, New Zealand).

Treatments were administered from a fixed point in the centre of square-frame PIT antennas at the upstream end of each subchannel (figure 1). PIT antennas were tuned to a detection sensitivity of roughly 0.3 m from the edges. Scan frequencies were programmed to 3 scans s^−1^. Treatments included (i) 3kPZS (5×10^−13^ M) administered into one subchannel (methanol into the adjacent subchannel), (ii) larval extract (5×10^−14^ M PADS) administered into one subchannel (methanol into the adjacent subchannel) and (iii) 50% methanol (in de-ionized water) into both subchannels for vehicle controls. Molar concentrations were the estimated final concentrations in the activated subchannel of the stream.

### 3.4 Data analysis

The dependent variables, proportions that moved upstream for 205 m to the channel confluence, were arcsine transformed and examined for violations of assumptions of normality and variance homogeneity using the univariate procedure in SAS (SAS Inc., Cary, NC, USA) before conducting statistical analyses. Upon observing no violations of the assumptions, analysis of covariance (ANCOVA, *α*=0.05) was used to test which explanatory variables influenced the upstream movement of pre-spawn female sea lamprey. The explanatory variables tested were (i) treatment (fixed), (ii) date (including year: random), (iii) stream temperature (fixed), (iv) rate stream temperature decreased per trial (fixed), (v) time when trial was conducted (fixed), and (vi) the number of animals released per trial (fixed). The final model was selected based on residual log likelihood (LogLik) and weighted AIC (*w*_*i*_) values [15]. The difference between the model with the lowest AIC and each additional model presented was calculated using equation (3.1):

3.1 $$\Delta_{i}={\mathrm{AIC}}_{i}-{\mathrm{AIC}}_{\min},$$

where Δ_*i*_ is the difference between the best fitting model (AIC_min_) and each model (AIC_*i*_). The normalized relative likelihood values were calculated using equation (3.2):

3.2 $$w_{i}=\frac{\exp(-0.5\times \Delta_{i})}{\sum_{r=1}^{R}\exp(-0.5\times \Delta_{r})},$$

where *w*_*i*_ is the weighted AIC value (Akaike weight) determined by dividing the relative likelihood of each model $\left(\exp(-0.5\times \Delta_{i})\right)$ by the sum of relative likelihoods of all models.

Differences of least-squares means (two-tailed *t* test, *α*=0.05) of the proportions of animals moving upstream per trial were examined at the lower (*q*_1_), median (*q*_2_) and upper (*q*_3_) quartiles of temperature across treatments (SAS). Quartiles in figure 1 were determined by first dividing the temperature data into two halves at the median (which was itself calculated as a mean of the two middle data). The median of the lower half of the data (lower quartile) and the median of the upper half (upper quartile) were then calculated. To determine whether pre-spawn conspecifics showed a more proximal preference towards treatments, we used logistic regression with a binomial distribution (R v. 2.11.1, Vienna, Austria) to examine (i) total number that moved upstream to the confluence of the subchannels, (ii) total number that entered the subchannel activated with each treatment, and (iii) total number in the treatment channel that entered the square administration point. Details of similar statistical analyses have been described [10,12]. No signs of nonlinearities or overdispersion were observed. All responses were compared with those of methanol control trials.

## 4. Results

### 4.1 Movement upstream

**Figure 2.**
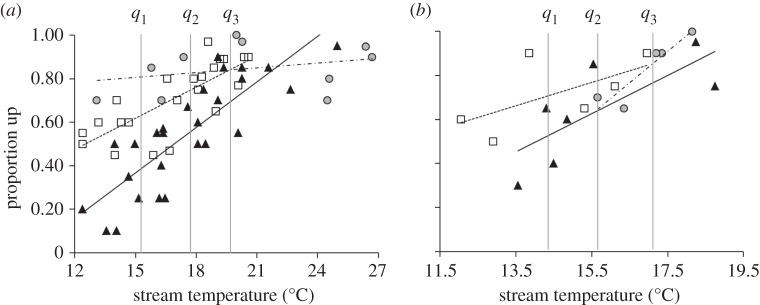
Upstream movement of pre-spawn female (*a*) and male (*b*) sea lamprey. Squares, trials where 3kPZS was administered to the stream (dashed regression line, females *R*^2^=0.59, males *R*^2^=0.38); upward triangles, trials where 50% methanol (control) was administered to the stream (solid regression line, females *R*^2^=0.68, males *R*^2^=0.57); and filled circles, trials where larval extract was administered to the stream (dotted-dashed regression line, females *R*^2^=0.10, males *R*^2^=0.85). *q*_1_–*q*_3_=the lower, median and upper quartiles of temperatures. See Results and table 2, for statistical comparisons at each quartile of temperature.

Female responses to 3kPZS during 2009 and 2010 were consistent (*F*_1,7_=3.59, *p*=0.100) and were combined in the analysis. Data from males were analysed separately because they were tested in a separate stream with different dimensions. The following variables did not influence the proportion of migrating females moving upstream across trials in our experimental system: the rate of stream temperature decrease per trial (°C h^−1^), time of trial (early or late) or the number of immature adult females released per trial (20 or 30) (ANCOVA: *F*_1,10_=0.97, *p*=0.348; *F*_1,12_=0.42, *p*=0.530; *F*_1,11_=0.01, *p*=0.919; *F*_1,12_=0.40, *p*=0.540, respectively), and were removed from the final model. The final model considered effects of treatment, stream temperature and their interaction as best explaining variability in the proportion of pre-spawn adult sea lamprey moving upstream (females: *F*_2,19_=9.36, *p*=0.002, males: *F*_2,3_=11.26, *p*=0.040; figure 2). Statistical fitness values were lowest in the final model (females: *logLik*=−25.3, AIC=−21.3, *w*_*i*_=0.22; table 1). This model was then used for analyses of male responses. *Post hoc* tests compared upstream responses at each temperature quartile. Overall, higher numbers of pre-spawn sea lamprey moved upstream in the presence of 3kPZS compared with methanol controls, specifically at the lower quartiles of temperature (approx. 15°C) as seen in figure 2 (females: *t*_19_=5.16, *p*<0.001, males: *t*_3_=1.22, *p*=0.310; table 2).

**Table 1.** Multiple effects, their interactions and fit statistics for each model examined for best explaining the variation in the proportion of sea lamprey that moved upstream across treatments.

effect(s)	interaction(s)	*logLik*	AIC	Δ_*i*_ AIC	$\exp(-0.5\times \Delta_{i})$	*w*_*i*_
odourant; temp	odourant×temp	−25.3	−21.3	0.00	1.00	0.22^a^
temp		−12.6	−6.6	14.70	0.48	0.10
	odourant×period	−4.8	1.2	22.50	0.32	0.07
	temp×period	−6.8	−0.8	20.50	0.36	0.08
odourant; temp; period	odourant×temp; odourant×period; odourant×temp×period	−5.8	−1.8	19.50	0.38	0.08
temp; period	temp×period	−5.8	0.2	21.50	0.34	0.07
	odourant×temp	−7.9	−3.9	17.40	0.42	0.09
odourant; period	odourant×period	−4.8	1.2	22.50	0.32	0.07
period		−1.6	4.4	25.70	0.28	0.06
odourant		−12.6	−6.6	14.70	0.48	0.10
date		3.7	7.7	29.00	0.23	0.05

^a^Final model selected with corresponding AIC and *logLik* values. Δ_*i*_ AIC and Akaike weights (*w*_*i*_) were calculated using equations (3.1) and (3.2). Relative likelihoods were calculated with the following equation: $\exp(-0.5\times \Delta_{i})$. Odourant refers to 3kPZS, methanol (control) or larval extract treatments. Period refers to early (20.00–23.00 h) or late (00.00–03.00 h) trials. Temp refers to stream temperature (°C).

**Table 2.** Treatment comparisons at the lower, median and upper quartiles of stream temperature in figure 2. Odourant treatments include methanol (control), synthesized 3kPZS (5×10^−13^ M) and larval extract (LE; 5× 10^−14^ M PADS). Italicized *p*-values indicate a significant difference (two-tailed *t*-test, *α*=0.05) in upstream movement between treatments at each quartile of the temperature range (figure 2).

	methanol versus 3kPZS		3kPZS versus LE		methanol versus LE	
females temperature (°C)	*t*_19_	*p*	*t*_19_	*P*	*t*_19_	*p*
*q*_1_ 15.5	5.16	<*0.001*	2.61	*0.017*	5.96	<*0.001*
*q*_2_ 17.9	4.59	<*0.001*	1.58	0.130	4.98	<*0.001*
*q*_3_ 19.7	2.76	*0.012*	0.36	0.726	3.13	*0.006*

	methanol versus 3kPZS		3kPZS versus LE		methanol versus LE	
males temperature (°C)	*t*_3_	*p*	*t*_3_	*P*	*t*_3_	*P*
*q*_1_ 14.4	1.22	0.310	1.86	0.161	1.16	0.330
*q*_2_ 15.6	0.66	0.558	0.89	0.439	0.49	0.657
*q*_3_ 17.1	0.46	0.678	1.57	0.214	1.51	0.229

### 4.2 Movement towards treatment channels

Pre-spawn sea lamprey did not prefer the 3kPZS treatment channel over the adjacent vehicle (methanol) channel across a full migratory season, yet both preferred the channel with larval extract over the adjacent vehicle channel. The same was true for within 0.5–0.25 m of the treatment source (table 3).

**Table 3.** Behaviour responses (percentage) of pre-spawn sea lamprey to treatments. Treatments: 3-keto petromyzonol sulfate (3kPZS, dissolved in 50% methanol), extracted water conditioned with larvae (larval extract, dissolved in 50% methanol) and control (vehicle, 50% methanol). Response variables: percentage swimming upstream (upstream), percentage entering the subchannel containing each treatment (treatment channel) and the percentage entering within proximity of the treatment source (treatment source). Different lower-case letters within each response variable within each sex indicate statistical differences (logistic regression; *α*=0.05).

sex	treatment	trials	released (*N*)	upstream (*n*)	treatment channel (*n*)	treatment source (*n*)
female	control	27	617	55% (342) a	50% (171) a	15% (25) a
female	larval extract	6	140	85% (119) b	86% (102) b	61% (62) b
female	3kPZS	22	489	71% (346) c	42% (146) a	18% (27) a
			*χ*^2^	59.56	67.03	53.21
			d.f.	2	2	2
			*P*-value	<0.001	<0.001	<0.001
male	control	7	140	64% (90) a	46% (41) a	76% (31) a
male	larval extract	5	100	83% (83) b	71% (59) b	90% (53) a
male	3kPZS	6	120	72% (86) a	55% (47) a	77% (36) a
			*χ*^2^	10.58	11.94	4.72
			d.f.	2	2	2
			*p*-value	0.005	0.003	0.095

## 5. Discussion

The male pheromone, 3kPZS, outweighed the effects of cold temperatures in modulating upstream movement of pre-spawning female sea lamprey. These results suggest that, in addition to functioning as a male-released sex pheromone [10], 3kPZS also functions as an indicator of the onset of spawning. Pre-spawn migrating sea lamprey showed a positive directional response towards proximity of the larval extract source, which remained consistent with previous studies [7,8]. Upstream movement at the lower quartile of temperature in males was not significant, yet the trend remained. Males, as releasers of 3kPZS, probably rely on a slightly different multitude of cues to establish nesting sites and begin to signal females.

Selective pressures favour the use of multiple sources of abiotic and biotic information to promote synchrony and reproductive success in animals that migrate long distances. Environmental cues such as temperature and stream flow [15], and conspecific cues such as larval odours and information regarding spawning ground size and quality (i.e. 3kPZS) may form a hierarchy of contradictory information that, when received in specific context, allows individuals to optimize timing of aggregation for reproduction. This study compares reliability of a particular abiotic cue and the conspecific signal 3kPZS, and provides insights as to why pheromone signals appear to be informative for migratory animals such as the sea lamprey.

## Supplementary Material

ESM 1 is an Excel file containing all raw data

## Data accessibility

Raw data are available in the electronic supplementary material.
